# ‘Pop-Up’ Governance: developing internal governance frameworks for consortia: the example of UK10K

**DOI:** 10.1186/s40504-015-0028-9

**Published:** 2015-09-28

**Authors:** Jane Kaye, Dawn Muddyman, Carol Smee, Karen Kennedy, Jessica Bell, UK10K

**Affiliations:** HeLEX Centre, Nuffield Department of Population Health, University of Oxford, Oxford, UK; Wellcome Trust Sanger Institute, Cambridge, UK; National Cancer Research Institute, London, UK; Nuffield Department of Population Health, HeLEX – Centre for Health, Law and Emerging Technologies, University of Oxford, Old Road Campus, Oxford, OX3 7LF UK

**Keywords:** Consortia, Genomics-research, ‘Pop-up’ governance, Networks

## Abstract

Innovations in information technologies have facilitated the development of new styles of research networks and forms of governance. This is evident in genomics where increasingly, research is carried out by large, interdisciplinary consortia focussing on a specific research endeavour. The UK10K project is an example of a human genomics consortium funded to provide insights into the genomics of rare conditions, and establish a community resource from generated sequence data. To achieve its objectives according to the agreed timetable, the UK10K project established an internal governance system to expedite the research and to deal with the complex issues that arose. The project’s governance structure exemplifies a new form of network governance called ‘pop-up’ governance. ‘Pop-up’ because: it was put together quickly, existed for a specific period, was designed for a specific purpose, and was dismantled easily on project completion. In this paper, we use UK10K to describe how ‘pop-up’ governance works on the ground and how relational, hierarchical and contractual governance mechanisms are used in this new form of network governance.

## Introduction

Information technologies have facilitated the development of networks, leading to new styles and forms of governance (Kersbergen & Waarden 2004). This is evident in the biomedical research context where research is increasingly carried out by large consortia that bring together interdisciplinary networks of researchers and institutions to focus on a specific research endeavour. Quite often, these projects are funded for a specific purpose over a limited funding period, and so are working to tight deadlines which require clear management, coordination and cooperation to achieve the goals of the project. Research must progress according to the agreed timetable, and allocated finances must be spent according to the approved grant submission. In response to these demands, new forms of network governance have developed to deal with the potentially contentious issues arising from the research activities of the consortium, such as publication moratoria, management of incidental findings, data access, as well as being accountable to external bodies. We are calling this new form of network governance ‘pop-up governance’, because it is established for a limited duration and purpose, and is dismantled once the project ends and it is no longer needed.

**Fig. 1:**
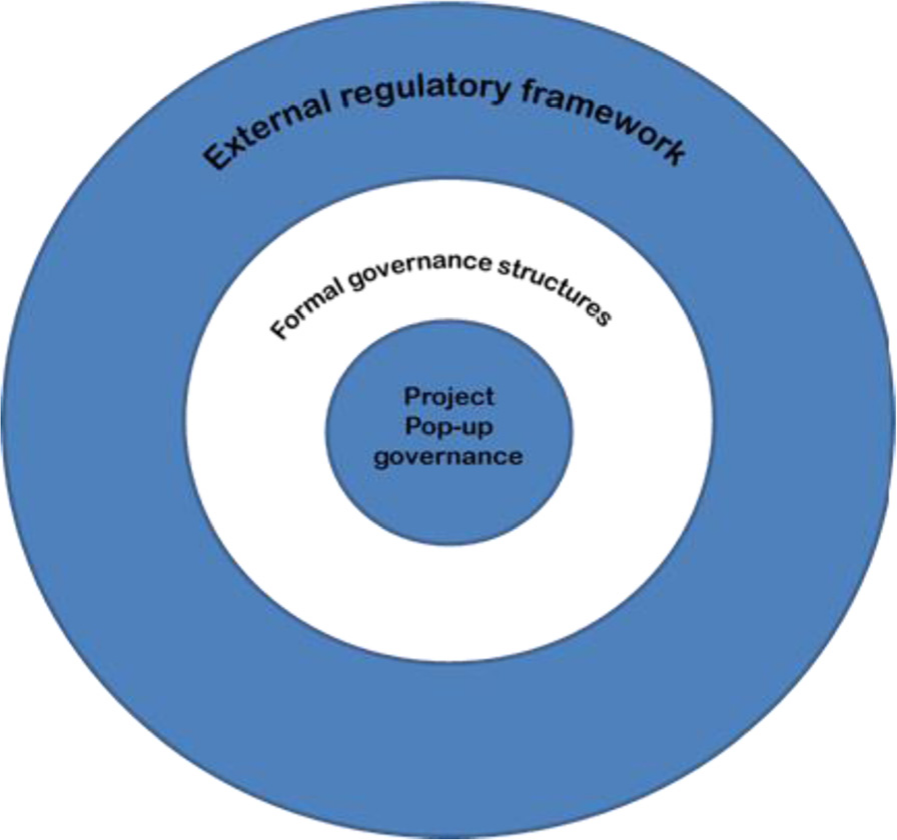
Features of ‘Pop – Up Governance

The system of committees and processes developed in the UK10K project (2010 – 2013) 1 is an example of ‘pop- up’ governance. The UK10K consortium was initiated and led by the Wellcome Trust Sanger Institute (WTSI) and was funded to establish insights into rare genetic diseases with the sequence data generated from the project used to establish a community resource. The purpose of this paper is to describe some of the features of this new form of research governance, which is found in interconnected research networks. We will use the UK10K project as an example of how ‘pop- up’ governance works in practice and show how it combined the features of networks with relational, hierarchical and contractual governance mechanisms. In the final section of the paper we will analyze some of the key components that are needed to make a ‘pop- up’ governance structure effective.

### Network governance

There has been a considerable amount of research on the organizational practices and arrangements that are network-like in form (Powell 1990). Networks have been defined as “groups of three or more legally autonomous organizations that work together to achieve not only their own goals but also a collective goal” (Willem & Gemmel 2013; Provan & Kenis 2008). Networks have been conceptualised as pluri-centric forms of governance, in contrast to multi-centric (market) and uni-centric (state / firm hierarchy) (Rhodes 2000). They are considered to be self-organizing, and to “resist government steering, develop their own policies and mould their environments” (Rhodes 2000: 61). They are characterised by an exchange of resources and negotiations and by game-like interactions “rooted in trust and regulated by the rules of the game negotiated and agreed by network participants” (Rhodes 2000: 61). While networks are diverse, there is sufficient generality to identify common characteristics but also to identify the circumstances that promote and sustain networks. The characteristics of networks identified in a number of studies are strong relationships (Willem and Gemmel 2013); interdependent experts (Powell 1990); collective working (Hertel et al 2000); groups with strong identities (Simon et al 2000); and the importance of a common goal. Many of these characteristics have been identified in the field of biomedical research by other scholars (Joly et al 2012; Dyke and Hubbard 2011; Fortin et al 2011; Field et al 2009; Tenopir et al 2011) and were evident in the ‘pop-up’ governance structure of the UK10K project. However, there have been no studies that have gone beyond identification of these features to demonstrate how they work as regulatory mechanisms in the governance structure of research projects.

There has also been considerable debate as to the type of governance mechanisms - relational, contractual and hierarchical - that lead to the most effective networks, and whether these elements are evident in all kinds of networks (Entwistle et al 2007; Herranz 2008). In their study of healthcare networks, Willem and Gemmel found there were combinations of hierarchical, contractual and relational governance mechanisms in use within healthcare networks. Relational governance ‘refers to co-ordination based on trust, reciprocity, and common norms and values that are embedded in the relationships between the partners in networks’ (Willem & Gemmel 2013). Although relational governance is traditionally thought to be the primary governance mechanism in networks, the studies in health care suggest that this might not always be the case (Willem & Gemmel 2013). For instance, Willem and Gemmel found that some of the contractual and pricing governance mechanisms that are typical of markets (Ferguson et al 2005) are also evident in healthcare networks. The contractual mechanism refers to the extent to which the collaboration is detailed and formalized in contracts.

Willem and Gemmel also found that hierarchal governance mechanisms were evident in healthcare networks, as collaborations were coordinated by one partner or a representative who had authority over others. This was in accordance with Milward and Provan’s research, (Provan & Milward 1995) that showed in networks where the government participates to provide services, such as mental health services, a hierarchical relationship might arise between the funding and controlling governmental agencies, and the non -profit organizations providing the services. The governance structure of the network can be deliberately chosen to give certain partners in the network more or less power, e.g. granting sufficient power to the financing government. Therefore, according to Willem and Gemmel, “Certain combinations of governance structure, governance mechanisms and network attributes lead to a more effective network configuration” (Willem & Gemmel 2013: 231). They found balanced combinations made an effective health care governance network. However, Willem and Gemmel have not as yet published a description of what these governance mechanisms might consist of. There has also been no analysis of this kind in regard to research networks, as their work focused on healthcare delivery networks.

The importance of this paper is that it articulates the concept and features of ‘pop-up’ governance that is found increasingly in biomedical research. The paper notes the existing research conducted by Lewis et al (2014) and the ‘pop- up clinic’ and contributes a description of the characteristics of network governance that have been identified in the literature that can be found in ‘pop-up’ governance and are exemplified by the UK10K project. By building on the work of Willem and Gemmel and their observations of clinical networks, we describe how the hierarchical, relational and contractual mechanisms are enacted in a research network, and in doing so extend the literature in this area.

### What is ‘pop- up’ governance?

The governance structures that have been developed for research consortia can be described as a form of network governance. In genomics, the lifetime of these structures is usually for the duration with the funding which accords with the time needed to deliver the specific goals of the project. ‘Pop- up’ governance, while transitory, is something more than just good project management or team organization. It can only succeed because there are other more formal governance structures around the project that support it and enable this ‘pop- up’ governance structure to be established and to function. Although they are of fixed duration, these governance structures are designed for optimum productivity as well as being structures that are reflexive and responsive to the many issues that arise as the research is initiated, coordinated and executed (Laurie 2011).

As a structure, ‘pop-up’ governance depends upon a complex blend of trust and respect for professional integrity and the ability of the project members to carry out their allocated project tasks, combined with more formal hierarchical mechanisms such as committees, procedures and guidelines, to deal with the potentially contentious issues arising from the research. Ways to manage access to samples and data are built into the project design to meet legal requirements and to ensure that the samples and data flow to the right people at the right time. External regulatory requirements must be met, such as the Human Tissue Act or, when dealing with personal data, adherence to the requirements of the Information Commissioner’s Office. Contractual mechanisms such as project agreements between the consortium collaborators and between the consortium and the funders are also essential for ‘pop- up’ governance.

It is this combination of formalized project committees and professional relationships that bring in relational, hierarchical and contractual elements that distinguishes ‘pop- up’ governance from good project or team management. While many of the issues around virtual team management that are discussed in the literature (Hertel *et al*^2000^) have a resonance with the governance structures of genomics consortium, this does not explain all of the dimensions of ‘pop- up’ governance, which also has a formal governance dimension. This feature of these consortia consists of a hierarchical management and administrative structure with delegated authority to committees, individuals or specialists, such as the Project Manager, or regulatory and policy advisers. These committees consist of individuals with specific expertise and knowledge that can deal with the issues that are central to achieving the objectives of the research project, such as publication moratoria, incidental findings and data access. Underpinning these committees are more relational mechanisms that are features of networks. The fact that these issues are also of importance to the external regulatory bodies, such as research ethics committees, funders and the host institution, provides another reason for ensuring that they are dealt with through a formal governance structure in a credible, transparent and accountable way.

Crucially, ‘pop- up’ governance can only exist because there is an external regulatory framework in place that supports it and from which ‘pop up’ governance emerges for the period that it is needed. This external regulatory framework can be described as a system of polycentric governance. Such regulatory regimes are those in which the state is not the sole locus of authority, or indeed in which it may play no role at all. They are marked by fragmentation, complexity and interdependence between actors, in which state and non-state actors are both regulators and regulated, and their boundaries are marked by the issues or problems which they are concerned with, rather than necessarily by a common solution (Black 2001). Within biomedical research there are a number of organizations within the UK that have a role in regulating this system of polycentric governance (Kaye et al 2012). In relation to genomics consortia, the key bodies are the research ethics committees (RECs) whose approval must be obtained before new research commences under NRES guidelines; the funders whose data sharing policies require that data must be shared widely; the online data repository where the sequence data is stored (in UK10K, the European Genome-phenome archive (EGA)) and the institutions that provide a safe working environment and are ultimately responsible for the conduct of their employees. However, all of these bodies give considerable latitude to researchers as to how research projects are run and carried out (Kaye & Hawkins 2014). This is particularly the case in research consortia in genomics where new policy, procedures, and practices in the form of ‘pop- up’ governance have been developed. These external bodies will provide valuable expertise when needed and therefore there is a flow of information between the ‘pop- up’ governance structure and the external governance bodies. However the basis of interactions relies on a complex blend of relational mechanisms with people working together, supported by contractual mechanisms such as project agreements, and researcher ethics committee approvals (Figure 1).

**Box 1 Features of ‘Pop- Up’ Governance**

**Table:** 

1. Has a limited life span
2. Is focussed on a specific purpose
3. Professional relations underpin the governance structure
4. Clear and decisive leadership and management
5. Expertise that can be utilised for specific tasks and committee functions
6. Clear committee hierarchy
7. Effective administration
8. Situated within an existing external governance system
9. Use of contractual mechanisms to underpin network activities

### The UK10K project

‘Pop- up’ governance has worked particularly well for the UK10K project^2^ and enabled the project to reach its objectives, despite this being a complex task, with a large number of contributing partners, all with different expertise and interests. The specific aims of the project were to improve understanding of the role of low frequency and rare genetic variants in health and disease, and to provide a data resource that could be used on a long term basis by the research community. The governance structure developed for UK10K had to be robust and able to adapt as required to meet these aims, as the tasks changed over time. At the time of writing, UK10K was Britain’s largest genomic sequencing consortium, having been awarded a £10.5 million Strategic Award by the Wellcome Trust.^3^

The main purpose of UK10K was to sequence the DNA of close to 10,000 people. To obtain this number of samples required input from a number of other studies where samples had already been collected or where there was easy access to patients to obtain new samples. Two well established cohort studies in the UK; TwinsUK and the Avon Longitudinal Study of Parents and Children (ALSPAC) provided four thousand of these samples, which were sequenced to approximately six fold coverage of their entire genome, generating a detailed sequence reference database connected to phenotypic and clinical data. Approximately six thousand samples were provided from existing collections spanning eleven different rare conditions or diseases to make up the total, and these were whole exome sequenced.

Patients included in the ‘disease’ arm of the project had already been diagnosed with a medical condition that was the subject of research, and all had been previously been recruited into other research studies prior to the commencement of UK10K. UK10K compared the sequences of individuals with a disease phenotype with those people from the cohorts group who had not been clinically diagnosed with the conditions being studied in UK10K, to try to identify rare and low frequency genetic variants associated with health and disease. It also provided an opportunity for researchers to be involved in a large, existing collaborative project that had been awarded funding which allowed sequencing to be carried out. It also provided an additional benefit of providing information that would become a unique research database for the research community as a whole. The data generated during the project was then made available to researchers outside UK10K via the EGA at the European Bioinformatics Institute (EMBL EBI), Hinxton, Cambridge, UK, which has the infrastructure to facilitate managed data access to data files.

As a collective endeavor, the UK10K project consisted of a number of stages and activities that had to be closely monitored, overseen and/or reviewed during their implementation, as would be expected for a sequencing project demonstrating best practice. A significant logistical challenge for the project was coordinating the activities of in excess of forty collaborators, from different disciplinary backgrounds, and working in different institutions across the country. To ensure that the project did not overrun or fail to achieve its sequencing target, it was crucial that strict deadlines for submitting samples to the project were adhered to by participating collaborators so that delays to the sequencing pipeline at the Wellcome Trust Sanger Institute (WTSI) were minimized.

To facilitate the smooth and efficient entry of DNA samples into the sequencing pipeline a number of regulatory and ethical requirements had to be met first, which also ensured the project was carried out to high standards of best practice. This began with a rigorous check that the existing consents and/or research ethics approval attached to the samples also permitted sequencing in a project such as UK10K, and the deposition of data in the EGA. Additionally, the fact that some of the disease phenotypes included in the project were rare or uncommon potentially increases the risk that some patients might have been re-identified and required extra safeguards in data handling. To minimise this risk, consideration of the type of data deposited, careful monitoring of data release and subsequent uptake by the community was essential, and had to be included in any governance framework for the project.

### The UK10K governance structure

From the earliest pre-planning stages of the UK10K project there was a clear need for defined, internal governance mechanisms that would enable project activities to be co-ordinated and carried out efficiently and effectively. In particular: meeting the timetable for sequencing through the Sanger Institute pipeline. Committees were established to oversee potentially contentious issues for the consortium and provided a mechanism for resolving these issues in a fair and transparent way. In doing so, this governance structure also provided a credible system of accountability for external funders and institutions. The set of committees that were established were hierarchical in nature, as some individual members of the consortium were delegated to make decisions that would affect the activities of other members. However, these committees only had credibility and legitimacy because they relied on the relational aspects of the consortium - the trust, reciprocity and common norms and values that were found in the relationships between collaborators in the UK10K project.

The formation of the Management Committee (MC), Ethical Advisory Group (EAG), the Data Access Committee (DAC), and the Publications Committee (PC) are examples of hierarchical mechanisms being used in a distributed network. The members of each committee were selected on the basis of their expertise and ability to commit sufficient time and resources as required for the successful running of the project. Some individuals, such as the Project Manager served on more than one committee, which was useful in those instances where committees had to work closely together to complete specific tasks and meet research deliverables. Each of these governance committees had delegated decision making responsibilities for designated activities within the consortium, and some went on to develop project policy to further guide decision making.

#### a) Management committee

The UK10K project had a clearly defined management hierarchy of committees and individuals, with the Management Committee (MC) being the single body empowered with sufficient authority (and ultimate responsibility) for directing the project to achieve its goals. It consisted of leaders from each of the research teams and all the co-applicants to the grant application, which had the effect of enabling transparent decision making but also keeping the project focussed on its deliverables.

The MC was identified at the grant application stage, and remained in place throughout the lifetime of the consortium. The MC took ultimate responsibility for the planning and execution of the project, and all other committees were required to report back to the MC on a regular basis, via appropriate channels. The committee approved all decisions relating to the fundamental delivery and quality of the research, and was the final arbitrator on any contentious issues arising during the project’s lifetime. Functioning as a single voice within a consortium of more than one hundred individuals, this body was accountable to the funders and the WTSI, for UK10K’s activities. However, the clinicians collecting samples were responsible to the regulators, such as the Human Tissue Authority for the collection of samples and relevant professional bodies such as the General Medical Council (GMC).

As the central governing executive for UK10K, the MC was led by the Principal Investigator (PI) and assisted by the Project Manager (PM). The PM oversaw and where necessary executed the MC’s decisions by working closely with the other committees and consortium members. The rest of the MC comprised of at least two representatives from each of the research groups working with DNA collections: cohorts, neurodevelopmental disorders, rare diseases and obesity; as well as representatives from the groups leading statistical analyses, ethics and production (sequencing and data delivery) in the project. The MC drew on the expertise and knowledge of the specialist committees answerable to it within the project and regulatory and policy advisers working within WTSI when making decisions. Also on the MC were the Production Chairs, who held a particularly pivotal role in the project, as they were ultimately responsible for overseeing the sequencing pipeline and delivering project data of a quality that was useful to other members of the consortium. Initially, MC meetings took place every two weeks in order to ensure a rapid response to any emerging issues and to set up appropriate mechanisms to move the project forward. Midway through the project when data generation was well underway and relatively problem free, such frequent meetings were unnecessary and the MC only convened once a month until the final stages of project, when the MC resumed its fortnightly meetings to ensure that all deliverables would be achieved on time and to budget.

#### b) Ethical advisory group

Whilst the consortium quickly identified certain production and process related issues that were likely to arise during the operation of the project, other issues were felt to be more difficult to address. To tackle these, the Ethical Advisory Group (EAG) was created to provide guidance on any ethical and legal issues emerging in UK10K. The EAG consisted of two co-chairs, eight UK10K researchers, a regulatory and a policy advisor and three external members, one of which represented a consortium of patient groups. One of the key documents produced by the EAG was the Ethical Governance Framework (EGF).^4^ This document sought to address some of the key ethical and legal issues that were likely to arise both before and during the project. These included:

1. Regulatory requirements and REC approvals;
2. Informed consent and the process of withdrawal from the project;
3. A management pathway for feeding back results to participants; and
4. Data access.

A scoping exercise was initially carried out to capture the wide ranging differences in donor consents and REC approvals which had been previously obtained by sample custodians holding pre collected samples from other studies, particularly referring to sequencing, the feeding back or otherwise of findings and data sharing. These had previously been obtained by sample custodians holding pre-collected samples for other studies. As these samples would be subsequently used in UK10K,the EAG were interested in recommendations concerning consent and REC approvals that needed to be included in the EGF. The EAG ensured that the requirements of the external regulators, the research ethics committees, were met and anticipated by all members of the consortium. If this had not been coordinated through a formal structure it would have been harder to meet the regulatory requirements within the timetable of the project.

When drafting the EGF document, members of the EAG drew on existing governance frameworks found in other projects, such as the UK Biobank and the International Cancer Genome Consortium. The EGF outlined ethical principles to which all researchers within UK10K must adhere, but afforded some flexibility to how those principles could practically be achieved. It ensured that samples used in the project had appropriate donor consent and/or REC approval attached to them, and this included approval for sequencing, deposition of data in an electronic archive and subsequent data sharing. The EGF was reviewed by all principle investigators within UK10K, then later by four external reviewers and posted on the project website. The EAG focused on particular issues that arose in the project, which were specific to meeting the project objectives, and had sufficient expertise to deal with them in a way that would meet the standards and concerns of external regulators. In doing so, this group focussed on addressing the contentious issues for the consortium, such as incidental findings, where there was no well- established procedure to draw upon. The management pathway for the return of pre-defined clinical results or incidental findings (Kaye and Hawkins 2014), to some participants, was developed because the extensive sequencing undertaken in UK10K meant it was highly likely that researchers would discover variations that could have health or reproductive significance for the participant. It was decided that these would only be returned if they were linked to the disorder which was the focus of the original study into which participants had been recruited.

#### c) Publications committee

The Publications Committee (PC) was established to help protect the first publication rights of UK10K’s sample custodians and data generators by overseeing a publication moratorium, and to ensure that project data used in publications (written by consortium collaborators) was correctly and appropriately acknowledged. Publications are very important to academic research communities - they are one of the metrics used to assess career progression - and also establish and maintain a scientific team’s reputation. Therefore, authorship can be a very contentious issue within a consortium, and who has access to data is important as this could determine the basis for authorship for papers based on UK10K data. The issue of publication moratoria arose during development of the project’s data access agreement at the start of the project, to balance the benefits of rapid data deposition in the public domain, with first publication rights of the data producers - as it was felt that it would be unfair for people to produce sequence data and then potentially be undercut on publishing a finding because they did not have the time to produce and analyse the data at the same time. It was agreed by the MC that a publication moratorium lasting one year struck an appropriate balance between making data available as soon as possible to researchers outside of the consortium, whilst giving UK10K researchers a reasonable timeframe in which to publish their own research findings. This moratorium period was also in line with the policies of previously funded Wellcome Trust projects such as the Wellcome Trust Case Control Consortium (WTCCC).

Members of the PC were nominated at the start of the project and endorsed by the MC. The committee comprised of the PM and a number of individuals who were also MC chairs, some based at the WTSI, and some based off site. This was an important balance; although WTSI was the leading institution in the project, the committee responsible for deciding the publication of research findings was not dominated by the same institution. As previously mentioned, an advantage of committee overlap was that it encouraged a regular dialogue between the PC and the MC. Any publications or documentation produced within the consortium using UK10K materials and citing the project had to be approved by the PC prior to submission, and this included all posters, abstracts and publications. The PC did not convene in person, but instead received documents by email (circulated by the PM). The PC required a minimum period of 1 to 2 weeks’ notice to review documents.

#### d) Data access committee

Not all of the committees were established internally in the project- the consortium used an existing external Data Access Committee to oversee applications from outside the project for access to the project generated information. This is an example of the consortium drawing upon an existing regulatory structure to help it resolve a potentially contentious issue and potential conflicts of interest. Due to the detailed and potentially identifiable nature of the sequence information generated in UK10K, it was decided from the outset that the data would only be made available to third party researchers under the terms of a managed access policy. This was to comply with the Wellcome Trust principles of sharing data,^5^ whilst still protecting the interests of both UK10K researchers and privacy of participants. Rather than set up its own DAC, the UK10K project drew on the pre-existing DAC already operating out of the Wellcome Trust (WT), London, to formally approve access to UK10K datasets held in the EGA. The WT DAC was already well established and highly competent at interpreting applications to use data. Crucially the DAC was independent from UK10K with no conflict of interest in the approval or rejection of applications.

The PM and the UK10K Production Team worked closely with the EGA to facilitate a rolling release of data to the EGA and, therefore, on to approved third party Data Users as and when the data was ready. In this way the community benefited from rapid access to UK10K data, rather than having to wait until the end of the project. To apply for access, researchers outside of UK10K had to download and complete a Data Access Agreement (DAA), designed specifically for the project.^6^ This would then be submitted to the PM as publicly designated point of contact within the project. After logging the application on the project wiki (allowing the PM to monitor the status of all DAAs through to the point of DAC approval), the PM would then triage applications (ensuring full and correct completion), before sending onto the DAC for final, formal approval. The DAC then notified the EGA and researchers were given access to the sequence data under the EGA’s managed access system. This is an example of where the consortium utilised the expertise of external bodies to help meet the objectives of the project, and enabled a system of accountability and oversight to be implemented that drew upon expertise that did not exist in the consortium.

### What are the key features of this governance framework?

The ‘pop- up’ governance system implemented in UK10K had a number of key features that enabled it to function as well as it did, nestled within an external regulatory and ethical framework. Routine and contentious issues alike could be resolved in a fair and transparent way between the involved parties, and the system was structured in such a way that decision making was documented (and/or minuted) and could be held accountable to the project’s funders. This structure was able to unite people from different disciplines and localities under a common banner of the project, while still allowing them the flexibility to use their professional expertise and judgment to carry out the tasks allocated to them. In this way, the project drew on a combination of relational, hierarchical and contractual governance mechanisms to enable the deliverables to be met. The key features of pop- up governance are: a common purpose, interdependency, a formal committee structure and an existing external governance environment.

#### a) Common purpose

A number of studies have investigated the theory of collective action and motivational gains, to investigate network relations. Collective action relates to specific behaviours designed to promote the welfare of one’s group (Tropp & Brown 2004). Group members act as representatives of their group in order to improve the situation of their entire group. Thus, collective action is understood as being guided by one’s self-representation as a group member and intended to bring about a change for one’s group as a whole (Kelly & Breinlinger 1996; Simon & Klandermans 2001). Simon et al. (2000) propose that when group identities become salient, there will be a shift in focus from the individual to the group, such that collective action will be motivated primarily by concern for and obligation to one’s group, and less by egoistic concerns of the individual. There is evidence that greater identification is associated with greater support for collective action*:* an inner sense of obligation to one’s group may underlie relationships between group identification and a willingness to engage in collective action for one’s group (Simon et al 2000).

Collective action and common purpose were key features of the consortium. This was strengthened by the fact that members had a common background; the more homogeneous the groups, the greater the trust and hence the easier it is to sustain network like arrangements (Simon et al 2000). This kind of relational governance (as opposed to contractual or hierarchical governance) enabled coordination based on trust, reciprocity, and common norms and values that were embedded in the relationships between the partners in networks (Willem & Gemmel 2013). Even though people had their own interests, the general ethos was that the project came first. At times this had to be reinforced through the committees but, in general, there was a shared assumption that the project aims came above individual concerns and research agendas. A strong driving force was that it was only by working together that everyone could achieve the benefits, as to sequence 10,000 genomes was beyond a single researcher’s capability. The clinicians supplied the samples from the patients they were treating and in return they had access to state of the art sequencing data for their patients which could assist with eventual treatment and diagnosis. They also had the benefit of being involved in a leading consortium. The WTSI brought state of the art sequencing technology and knowledge of running large projects, but they also had a research interest in analysing the sequence information. This common purpose overcame the geographical distribution of the consortium. Although the consortium members were dispersed in different institutions across the country, the geographical distance between project members did not impede the project. This was because there were strong motivational rewards for participation in the project, being part of a large, collaborative project that was establishing a sequencing resource for the country. This alignment was a strong incentive for collaboration, and could be seen as a relational governance mechanism that provided the basis for developing trust by establishing or intensifying the collaboration between the researchers in the project.

#### b) Interdependency

Studies involving team sports have proven that interdependency between individuals is a motivator for collective action; meaning that individuals work harder as a team than as individuals so as not to let other team members down (Messé et al. 2002). Furthermore, repeated interaction amongst individuals has powerful consequences; “when there is a high probability of future association, persons are not only more likely to cooperate with others; they are also increasingly willing to punish those who do not cooperate” (Axelrod 1984).

The UK10K project had very clear goals, and each member of the consortium was a member because of the role that they would play in achieving these goals. The sequencing pipeline meant that all of the member’s activities were dependent on each other and they had to coordinate their independent activities frequently. This alignment meant that the possibility of someone losing motivation or not knowing how their actions related to others was not possible. Another factor was the potential for reputational harm. This has been found to be a strong motivator in other network settings, where there is little separation of formal business statuses and personal social roles (Powell 1990). Thus, the relational aspects of a network can lead to strong compliance with project decision-making and prioritisation of the project objectives by collaborators. These relational elements were key to the aims of UK10K.

In the UK10K project, compliance was ensured by the project’s shared vision and to some extent, peer pressure. Individual members did not want to be perceived as the person that was slowing down progress. If an individual was not pulling their weight this was made obvious by the sequencing pipeline, which had to be carefully co-ordinated within the allocated sequencing time. Weekly MC meetings were an opportunity for performance review and accountability. They were also an opportunity to congratulate good work in the project. These meetings were transparent and the MC could flag up any issues and what needed to be done to address these. This internal monitoring encouraged the various parties to do their tasks and where necessary, to do them to a higher standard. For individual researchers their professional reputation was at stake if they let people down, which encouraged people to meet deadlines and make the project a priority in amongst their other work and research commitments.

This is in accordance with other studies where it was found that ‘persons mobilize additional efforts when they believe that own poor performance would inhibit other team members in their work’ (Hertel et al 2000; Messe et al 2002).

#### c) Committee decision making

The formal, hierarchical structure of committees in the UK10K project achieved two distinct goals. First, they enabled the project deliverables to be met, and second they enabled the contentious issues in the project around publications, ethical and legal issues and data access, to be addressed in a transparent way by peers. However, these hierarchical governance mechanisms were only successful because they depended upon relational aspects of the network, demonstrated by each of the committees within the UK10K project.

One of the key features of the UK10K governance framework was that by working together, the committees developed a web of governance that created a system of ‘checks and balances’ to ensure that project deliverables could be met. This meant that important issues of concern to members of the project and external funders and institutions, which had potential implications for research integrity and professional standing, were dealt with quickly and transparently.

This delegation also took the responsibility for decision making away from the hard pressed MC and placed it in the hands of people with expertise and knowledge. Powell has highlighted how “know-how; the demand for speed, and trust” are critical components of networks (Powell 1990). Therefore, the highly skilled, intellectual workforce of the UK10K consortium was particularly well suited to a network form of organization, but also provided the basis for effective committees.

The responsibilities of each of the committees were clearly defined from the outset, and shared across those who were willing and had the relevant expertise. This meant that there was sufficient expertise, delegation and interrelationships between the governance structures, to make decisions independent of the MC. For example, the work of the DAC was made easier because the PM conducted a preliminary review of all DAAs before forwarding them on. This review stage was easy for the PM to undertake because of her working knowledge of all the UK10K studies included in the project, and any restrictions on data use. In turn, the MC welcomed the role of the DAC in granting final approval for DAAs, as this allowed the MC to remain impartial in the matter of data access, and ensured that the process was not hindered or delayed.

This principle also applied to the approval of UK10K publications; having a dedicated publication committee ensured that proposed publications were quickly approved and submitted to journals, whilst still protecting the interests of the consortium. Having a fixed group acting as the PC ensured the continuity of policy regarding authorship. It also fostered trust as authors were reassured that their efforts would not be exploited by other team members (Harlow & Rawlings 2007). Overall, these structured committees enabled the project deliverables to be achieved within a very tight time frame, which was important in a project where resources were limited and required an efficient use of people’s time and expertise.

This interrelationship was possible because there were people that linked the various committee activities of the project together (Guzzo & Dickson 1996; Hertel et al 2003). For example, the Project Manager was an important member of the MC and the PC, and also played a key role in UK10K data management. As a designated point of contact for the project both within and outside of the consortium (and also to the public), the PM helped to coordinate activities between committees and across UK10K. This was also true of some of the key people who served on the various committees, were members of their disease sub-group, and lead a task. This enabled decision making to be fast and responsive, but also committees were aware of the decisions made in other fora of the project. People in these positions were aware of the different responsibilities that they had, and that as a representative of the project their decision making had to be appropriate to the committee they were serving on. Having committees gave a certain level of accountability and transparency. While this feature is not explicitly found in the literature, there is recognition that networks are based on complex communication channels and a key advantage is their ability to disseminate and interpret new information, resulting in new interpretations and ultimately new linkages (Guzzo & Dickson 1996; Hertel et al 2003).

#### d) External formal structures

The creation of the ‘pop-up’ governance structure was facilitated because it was supported by external formal structures and contractual mechanisms. The institutions in which researchers were based in provided the necessary working environment to meet many of the legal requirements and to support the research. It was these institutions that contracted with the funder, the Wellcome Trust by providing sponsorship for the project through the funding agreement, which established the legal obligations and responsibilities of all members of the consortium. It would not have been possible for the relationships to be supported and the project to exist without these institutional agreements. This structure of formal contractual governance mechanisms allowed the relational mechanisms of the research network to flourish. They also provided the support for the pop-up governance structure to be established and maintained.

As well as being supported by the external regulatory and ethical framework, the 'pop-up' governance structure also enabled the requirements of external regulators, ethical advisory bodies and WTSI internal policy to be met by providing a framework for enacting these requirements. Within the UK there are a number of regulatory bodies such as the Human Tissue Authority, the Information Commissioners Office and ethics advisory bodies, such as research ethics committees, that place requirements and/or ethical obligations on researchers. The ‘pop-up’ governance structure provided a mechanism to meet these requirements in a systematically and orderly manner for the consortium as a whole, rather than for individual researchers. Therefore, 'pop-up' governance could deal with potentially challenging issues through its committee structure, which were the same issues that were of concern to the institutions in the external regulatory environment. In this way the ‘pop-up’ governance structure enabled the consortium to operate as an effective whole to meet its own objectives while at the same time being able to demonstrate accountability to external regulatory bodies, institutions and funders, and uphold best ethical practice (Brownsword and Goodwin 2012).

## Conclusion

For projects such as UK10K, there are limited resources both in terms of expertise and time to establish governance systems, yet there are good reasons why such projects should have a responsible and accountable governance structure in place. The ‘pop- up’ governance structure that was developed for UK10K provided a system of accountability and transparency in decision making that achieved a number of goals. Firstly, it had the effect of promoting trust between the members of the consortium, but also provided a structure that inspired confidence from external institutions, funders, regulators and ethical advisers. It also provided an effective governance structure for meeting the project deliverables, complying with the legal requirements of regulators in the external regulatory environment and working to best ethical practice. To achieve this, a combination of hierarchical, relational and contractual mechanisms were used that draw upon the relational features of networks to be effective.

One of the features of this project is that all of the people involved had a shared vision of what had to be achieved and a commitment to achieving the goals of the project. The project was a success because the central aims of the project also corresponded with researcher’s individual research interests and goals. By working together, researchers achieved more than they would have by themselves or with a smaller partnership. The ‘pop-up’ governance structure united people from different disciplines and localities under a common banner of the project, while still allowing them the flexibility to use their professional expertise and judgment to carry out the tasks allocated to them. It had formal structures of decision making, such as committees with individuals with expertise who were well regarded for their professional knowledge or integrity, and were delegated by the consortium to sit on the committees. Underpinning the governance structure was a series of professional relationships based on trust, but also on the traditional scientific drivers of not wanting to be regarded as a weak member of the consortium. Therefore, 'pop-up' governance was heavily dependent not only upon good relationships between researchers, but also on strong leadership.

The benefit of the ‘pop- up’ governance system is that it fitted in within existing governance structures. This structure made it clear who was responsible for what, and the procedures were written down in policies which had the effect of promoting certainty and efficiency. This clarity in roles meant that everybody understood what their role was in the project and the streamlined structure also lead to benefits as it enabled scientists to do science, whilst still being involved in the governance structures. It also meant that everyone had to go through the same procedures and therefore decision making was more transparent, helping to eliminate favouritism. Such a system enabled problems to be anticipated as there were mechanisms in place to deal with routine issues but, also, unanticipated situations could be resolved efficiently. Having a governance system in place ensured that ethical and lawful research was supported through accountable and transparent decision making. This not only protected the integrity of the UK10K researchers and their institutions, but also had the effect of promoting trust between different members of the consortium.

In summary, fundamental to the successful acquisition, use and dissemination of UK10K sequence data and the undertaking of good and ethical science, was the support of a robust and flexible system of internal self-regulation, developed specifically for the project. The project governance system did not burden the project with unnecessary or inappropriate administrative checks and balances that could potentially tie up expertise and resources. The formal governance components of the UK10K project focused only on the important issues, such as best ethical practice, publications, data access, feedback of findings and accountability to funders and the sponsoring institution. All other issues were dealt with through the Management Committee, relying on the relational mechanisms of trust, co-operation and co-operation. The advantage of this internal governance framework was that it could address issues such as incidental findings, where best practice had not been established and there was no clear legal direction. The UK10K governance framework was designed to provide assurance and accountability to the researchers involved in the project, the funders of the project, the employing institutions, external regulators, ethical advisers and ultimately where necessary, the research participants themselves.

## Footnotes

1 UK10K Website, http://www.uk10k.org/. Accessed 5/7/2013

2 UK10K Website, http://www.uk10k.org/. Accessed 5/7/2013

3 See http://www.wellcome.ac.uk/News/Media-office/Press-releases/2010/WTX060061.htm

4 UK10K Ethical Governance Framework: http://www.uk10k.org/assets/ef_uk10k_v21.pdf Accessed 16 July 2013

5 Welcome Trust Statement on genome data release, http://www.wellcome.ac.uk/About-us/Policy/Policy-and-position-statements/WTD002751.htm

6 UK10K Data Access Application: http://www.uk10k.org/data_access.html. Accessed 16 July 2013
